# Sudden death due to the atrioventricular node contusion: Three cases report

**DOI:** 10.1097/MD.0000000000005688

**Published:** 2017-01-10

**Authors:** Wenhe Li, Lin Zhang, Yue Liang, Fang Tong, Yiwu Zhou

**Affiliations:** a Department of Forensic Medicine, Huazhong University of Science and Technology, Tongji Medical College; b Department of Pathology, The Central Hospital of Wuhan, Tongji Medical College, Huazhong University of Science and Techology, PR China.

**Keywords:** atrioventricular node contusion, blunt cardiac contusion, cardiac conduction system, posterior atrioventricular junction, sudden death

## Abstract

**Introduction:**

Atrioventricular node (AVN) contusion usually results in cardiogenic shock and arrhythmia and is a rare but fatal condition. The condition is difficult to diagnose and easily overlooked because it develops rapidly and is asymptomatic. We here report 3 cases that demonstrate blunt chest impact and hemorrhages of the posterior atrioventricular junction, eventually result in death.

**Clinical Findings:**

Autopsy and histological examination were performed on all cases. External inspection revealed bruises in the hearts and fractures in the sternum and ribs. However, histological examinations were conclusive and showed cardiac contusion on the surface of the posterior atrioventricular junction of the individuals, and the death was due to the AVN contusion. The position of the AVN on the heart surface is determined by detailed examinations via an autopsy and microscopic, both of which are critical in the certification of cause of death.

**Conclusion:**

The report is intended to raise our understanding and make forensic pathologists aware of the surface of the posterior atrioventricular junction.

## 1 Introduction

Cardiomyopathies, coronary artery diseases, and functional dysregulations are heart conditions that can lead to sudden death and are some of the core topics in forensic pathology.^[1–3]^ Over the past decades, blunt chest trauma with injury to the heart has become increasingly common, particularly as a result of severe crushing injuries to the thorax and the lower body.^[4]^ Blunt chest trauma results in myocardial contusion, myocardial rupture, valvular disruptions, and rupture of coronary arteries, which are the great vessels and the pericardium. Secondary to blunt chest impact, few cases associated with injury to the atrioventricular node (AVN) region and the bundle of His have been reported.^[5,6]^ Death caused by AVN contusion following blunt chest trauma and trauma to the vulnerable locations surrounding an AVN contusion have not been reported so far.

Herein, we report 3 cases that demonstrate blunt chest impact and hemorrhages of the posterior atrioventricular junction, eventually result in death. Autopsies of these cases revealed AVN contusion.

## 2 Clinical Findings

### 2.1 Case 1

**Figure 1:**
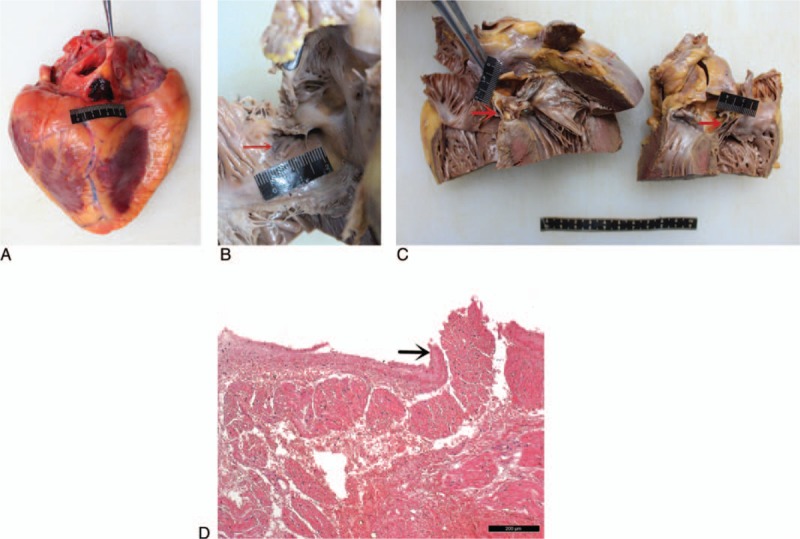
(A) A bruise was found between the right inferior vena cava and the right coronary sulcus of the posterior atrioventricular groove epicardium. (B) Hemorrhage was present on the leading edge of the coronary sinus from the perspective of right atrium (as the red arrow show). (C) The horizontal section showed the transmural hemorrhage that involved the coronary sinus (the red arrows indicate). (D) The laceration was seen on the lower edge of the coronary sinus (the black arrow indicate).

A 25-year-old man was brought to hospital after being unconscious and collapsed during a physical fight with someone. He was declared “dead on arrival.” Autopsy revealed an abrasion on the 5th and 6th intercostal space on the left side of the chest measuring 3.0 cm, and hemorrhages on the right pleura and its corresponding intercostal muscles. The heart was enlarged and dilated (weight 466 g) with an area of recent bruising measuring 2.0 cm × 1.5 cm between the right inferior vena cava and the right coronary sulcus of the posterior atrioventricular groove epicardium (Fig. 1A). From the perspective of the right atrium (RA), hemorrhage was present on the leading edge of the coronary sinus (Fig. 1B). Dissection of the right atrial wall showed the transmural hemorrhage that involved the coronary sinus (Fig. 1C). No lethal injuries were identified clinically or at the autopsy. Histological examination exposed infiltrating hemorrhages involving all layers of the orifice of coronary sinus, some necrotic myocardial cells with scattered inflammatory cells and a small amount of white thrombus attached to the corresponding subepicardial region. In addition to the foregoing lesions, a laceration was seen on the lower edge of the coronary sinus (Fig. 1D). There were no particular histological findings in other organs apart from slight congestion. No alcohol and drugs were detected. Consequently, it was considered that arrhythmia was induced by the AVN contusion and was regarded as the cause of death.

### 2.2 Case 2

**Figure 2:**
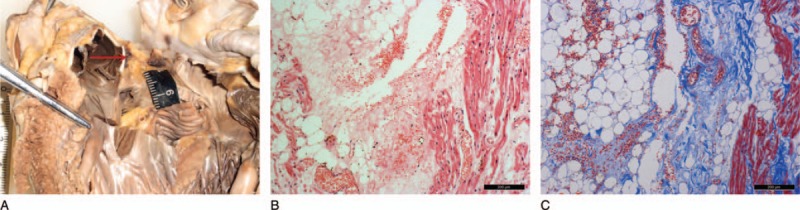
(A) Hemorrhage was seen between the left coronary leaflet and right coronary of the atrioventricular node (AVN) region (the red arrow). Hemorrhage was clear within the AVN via hematoxylin-eosin staining (B: HE stain, ×100). Hemorrhage was obvious within the AVN via Masson trichrome staining (C: Masson trichrome stain, ×100).

A 13-year-old boy was kicked on the chest and the right abdomen and lost consciousness, eventually died on arrival at a hospital emergency care unit. During the autopsy, there were 2 reddish abraded contusions of the left nipple, measuring 4.5 and 4.0 cm. The heart weighed 215 g, which was within the normal reference range, and hemorrhage measuring 0.6 cm × 0.2 cm was present between the left coronary leaflet and right coronary of the AVN region (Fig. 2A). Histological examination showed interstitial foci of hemorrhage, preferentially located around the AVN with broken myocardial fibers (Fig. 2B and C). The myocardial interstitium was also edematous and other organs disclosed no microscopic anomalies. Toxicological analysis was negative for drugs and alcohol. The death was due to the AVN contusion caused by blunt chest impact.

### 2.3 Case 3

**Figure 3:**
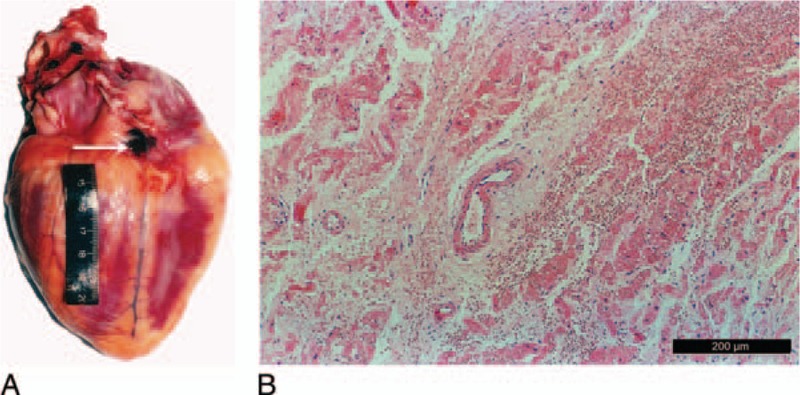
(A) Hemorrhage was found between the fossa ovalis and the coronary sinus orifice of the AVN region (the white arrow). Myocardial cells were divided into different shapes by the red blood cells (B: HE stain, ×100).

A 22-year-old male collapsed following an episode of being kicked on the head and chest. At the autopsy, there was bruising measuring 5.0 cm × 2.0 cm on the left side of the chest wall with incomplete rib fractures. Hemorrhage measuring 1.7 cm × 1.2 cm was found in the AVN region of the facies diaphragmatica cordis, preferentially located near the fossa ovalis and the coronary sinus orifice (Fig. 3A). On microscopic examination, the atrial muscle fiber revealed tension, separation, and necrosis surrounding an area of hemorrhage. Myocardial cells with cytoplasmic swelling and vacuolar degeneration were divided into different shapes by the red blood cells (Fig. 3B). It was considered that the death was attributed to the AVN contusion. In addition, toxicology screening was negative for common drugs and alcohol.

This study was approved by the Ethics Committee of Tongji Medical College, Huazhong University of Science and Technology, Wuhan, Hubei, China, and written informed consents were obtained from the family members of the patients.

## 3 Discussion

The AVN is an important region of the heart where the atrial myocardium inserts into the base of the ventricular mass.^[7]^ From the point of topographic endocardial landmarks, it is located at the base of the RA defined by the landmarks of the coronary sinus ostium and the septal leaflet of the tricuspid valve. It is described as a compact spindle-shaped network of cells arranged near the central fibrous body (CFB) connected to the bundle of His, both being responsible for the only physiological atrioventricular axis of conduction.^[8]^ Transverse section seems like a microconvex triangle and seems to be dilated in the middle region when viewed from the right side. The AVN is mainly supplied by the atrioventricular nodal artery (AAVN), which passes through the node and penetrates the CFB. The AAVN can have a right or left coronary artery origin, though the AAVN most commonly originates from the right coronary artery, that is, in 90% of patients.^[9,10]^ The distal segment of the AVN has a dual blood supply in 80% of human hearts from the same AAVN and the left anterior descending artery. It is an indispensable component of the cardiac conduction system axis and the only electrical conduit from the atria to the ventricles.^[11]^ Accordingly, the electrophysiological activity of the AVN can cause different degrees of atrioventricular block and result in cardiogenic shock and may occur when the AAVN is in disorder.

The examination of endocardium, epicardium, valves, and coronary arteries are routinely performed. However, an AVN contusion is often ignored because of coexistent injuries such as fractured ribs and pulmonary contusion. The chief complaints usually mask the manifestations of cardiac injury.^[12,13]^ As it is, cardiac contusion is one of the most common complications of blunt chest trauma that follows violent fall impacts, aggressions, and the practice of risky sports.^[14]^ Cardiac contusion happens in approximately 30% of severe blunt force trauma cases.^[15]^ The mechanism of cardiac injury involves a sequence of events beginning with direct impact to the chest wall with transmission of the kinetic force to the patient, and this results in compression of the heart between the sternum and the spine.^[4,16]^ The AVN is very vulnerable to damage when there is cardiac contusion.^[17]^ The mechanisms could be divided into 3 categories based on the type of force, namely compression injury, traction injury, and crush injury. Compression injury is mostly caused by direct impact and trends to act on the precordial region and the back leading to heart injury. The pressure put on the sternum and spine secondary to blunt chest injury makes the atria and ventricles more vulnerable.^[4]^ Traction injury often occurs with rapid deceleration that causes disruption of the atria from their junctions with the vena cava and pulmonary veins.^[18–20]^ Crush injury is caused by the rapid increase in compressive forces arising from the lower extremities and abdomen due to increased intrathoracic hydrostatic pressure, and this radiation of forces manifests in the form of transmission of the raised venous pressure directly to the atria resulting in injury to the heart.^[4]^ Cardiac injury may also be caused by severe changes in atmospheric pressure surrounding the body, as is commonly seen in victims of explosion.^[19]^ Because the right heart is quite superficial and more often affected than the left.^[21]^ Electrophysiological and structural abnormalities, promoting abnormal impulse formation and propagation, lead to dysrhythmias.^[22]^ And transient dysrhythmias may be observed caused by cardiac contusion after blunt chest trauma, such as mechanical or electrical dysfunction including high degree atrioventricular block and ventricular fibrillation, the mechanism is that ionic channels alterations and its effect on cardiac depolarization and subsequently cardiac death.^[23,24]^ However, rare clinical cases in the literature confirm that blunt chest trauma can cause conduction defects.^[25]^ This report highlights 3 cases of men who suffered a blunt force on the precordial region and died immediately. At the autopsy, rib fracture, and subcutaneous tissue and intercostal muscles hemorrhages could be seen. Moreover, they all experienced blunt impact on the precordium during their lifetime.

The report described a unique feature that hemorrhages of the right posterior atrioventricular junction surface and the corresponding inner surface with AVN contusions were determined by autopsies. In the 1st case, hemorrhage could be seen between the right inferior vena cava and the right coronary sulcus of the posterior atrioventricular groove in the epicardium. The corresponding region of cardiac chamber was the leading edge of the coronary sinus orifice. The AVN contusion in this may be considered a compression of injury type according to the scenario. When observed from the medial surface, clearly demarcated, broken down hemorrhage was found in the AVN region involving the epicardium and the underlying myocardial tissue. The contusions included the epicardial mesothelium, vessels, nerve, adipose tissue, fibrous tissue, and myocardium. In the 2nd case, hemorrhage was also identified between the root of the 3 tricuspid septal leaflets and the CFB. Histological examination revealed multiple myocardial contusions involved with the epicardium, myocardial layer, and endocardium. The AVN contusion can be attributed to the traction injury in this case. The AVN showed broken myocardial fracture, myocardial interstitial edema, and inflammatory cell infiltration around the contusion. In the 3rd case, hemorrhage was found on the AVN of the facies diaphragmatica cordis, preferentially located near the fossa ovalis and the coronary sinus orifice. The AVN contusion was caused by the crush injury mechanism. Neither the coronary artery nor the working myocardium showed atherosclerosis or any recent ischemic signs in the reported cases. It is concluded that the right atrioventricular junction contusion is likely to damage the AVN.

AVN contusion was diagnosed on the basis of an obvious history of trauma to the chest, back, and abdomen, the injured person died instantly. Moreover, to collect and analyze clinical materials is very important. Continuous monitoring systems may monitor patient's clinical course and device function, such as tele-monitoring (TM).^[26]^ The cardiac injury cases should undergo careful examination involving the heart and any other relevant clinical material should be gathered to identify cardiac contusion and heart disease. Furthermore, AVN contusion needs to be differentiated from other complications of blunt trauma, especially cardiac concussion and cardiac inhibition. A history of precordial region trauma and death within a short time period are the common characteristics of cardiac concussion and cardiac inhibition. The main feature of cardiac concussion death is characterized by little or no evidence of pathologic changes either grossly or microscopically to the heart.^[27]^ Therefore, it is easily ignored by clinicians. From the forensic pathologists point of view, cardiac concussion has been defined as sudden death from a seemingly trivial nonpenetrating blunt trauma to the chest, with no demonstrable intrathoracic injury and without histological evidence of injury that might induce sudden lethal dysrhythmias that results in death.^[28,29]^ Death from inhibition is generally regarded as an unexpected death from a slight stimulation or force that was imposed on certain parts of the body with abundant nerves. This force or subsequent stimulation results in cardiac arrest through nerve reflex in a short period and the autopsy of this individual cannot be used to make clear the cause of death. The cardiac autonomic nervous system consists of the parasympathetic and the sympathetic systems.^[30]^ The parasympathetic innervation of the heart originates from the medulla oblongata. Stimulation of the sympathetic branch exerts facilitator effects on function, increasing heart rate and myocardial contractility, whereas the stimulation of the parasympathetic branch exerts inhibitory effects that decrease heart rate and contractility. Vagus nerve belongs to the cardiac parasympathetic nervous system and has been known to play a predominant role on atrial pacemakers. The enhanced vasovagal reactions may cause the inhibition of normal heart activity and death when a blunt force acts on the chest. Sudden death due to vagal inhibition caused from a minor trauma has been previously reported.^[31]^ Specific morphological lesions could not be seen at autopsy in this case of cardiac inhibition.

As for the report, forensic pathology revealed no deadly disease, and toxicological analysis was negative for drugs and alcohol. It was worth noting that the reported cases all suffered from blunt impact to the chest and hemorrhages were seen between the RA and left atrium on the posterior aspect and at the base of the heart. Upon endocardial exposure, the transverse sections portrayed the super posterior region of the medial atrial wall which is superior to the coronary sinus orifice. Eventually, the victims were deemed to have died due to arrhythmia and cardiac arrest induced by the AVN contusions.

In conclusion, to our knowledge, this is the first report to determine the surface position of the AVN contusion. The examiners should pay more attention to the AVN when the posterior atrioventricular junction contusions after blunt force impact to the chest, especially to confirm cardiac contusion. In addition, the AVN contusion needs to be differentiated from other complications of blunt trauma using as a guide the process of the death, autopsy findings, and histopathological examinations. As is relevant from this report, our understanding about death associated with AVN contusion is of utmost importance to the forensic pathologists and clinicians to confirm sudden death due to the AVN contusion.

## Acknowledgements

The authors thank Project 81273336 supported by National Natural Science Foundation of China for the support. The authors also thank Dr Basil Arif and Dr Mohammed Mahmoodurrahman for correcting the manuscript.

## References

[1] Boriani G Valzania C Diemberger I Potential of non-antiarrhythmic drugs to provide an innovative upstream approach to the pharmacological prevention of sudden cardiac death. Expert Opin Investiga Drugs 2007;16:605–23. 10.1517/13543784.16.5.605 17461735

[2] Maujean G Tabib A Malicier D Sudden death due to a cystic atrio-ventricular node tumour. J Forensic Leg Med 2010;17:437–8. 21056880 10.1016/j.jflm.2010.08.009

[3] Myerburg R Kessler K Castellanos A Sudden cardiac death. Structure, function, and time-dependence of risk. Circulation 1992;85 (1 Suppl):I2–10. 1728501

[4] Turan AA Karayel FA Akyildiz E Cardiac injuries caused by blunt trauma: an autopsy based assessment of the injury pattern. J Forensic Sci 2010;55:82–4. 19895542 10.1111/j.1556-4029.2009.01207.x

[5] Zhu B-L Fujita MQ Quan L A sudden death due to cardiac conduction system injury from a blunt chest impact. Leg Med 1999;1:266–9. 10.1016/s1344-6223(99)80049-2 12935480

[6] Zerbo S Maresi E Portelli F Death of a 23-year-old man from cardiac conduction system injury through a blunt chest impact after a car accident. Egypt J Forensic Sci 2014;4:137–9.

[7] Anderson RH Ho SY Anatomy of the atrioventricular junctions with regard to ventricular preexcitation. Pacing Clin Electrophysiol 1997;20:2072–6. 9272512 10.1111/j.1540-8159.1997.tb03631.x

[8] Kurian T Ambrosi C Hucker W Anatomy and electrophysiology of the human AV node. Pacing Clin Electrophysiol 2010;33:754–62. 20180918 10.1111/j.1540-8159.2010.02699.x PMC2889145

[9] Stambler B Rahimtoola S Ellenbogen K Pacing for atrioventricular conduction system disease. Clinical Cardiac Pacing, Defibrillation, and Resynchronization Therapy 3rd ed Philadelphia:Saunders; 2007 429–30.

[10] Sow M Ndoye J Lo E The artery of the atrioventricular node: an anatomic study based on 38 injection-dissections. Surg Radiol Anat 1996;18:183–7. 8873331 10.1007/BF02346125

[11] Dobrzynski H Anderson RH Atkinson A Structure, function and clinical relevance of the cardiac conduction system, including the atrioventricular ring and outflow tract tissues. Pharmacol Ther 2013;139:260–88. 23612425 10.1016/j.pharmthera.2013.04.010

[12] Esmaeilzadeh M Aortic valve injury following blunt chest trauma. Res Cardiovasc Med 2014;3:e17319–17319. 25478541 10.5812/cardiovascmed.17319 PMC4253800

[13] Esmaeilzadeh M Alimi H Maleki M Aortic valve injury following blunt chest trauma. Res Cardiovasc Med 2014;3:3. 10.5812/cardiovascmed.17319 PMC4253800 25478541

[14] Holanda MS Domínguez MJ López-Espadas F Cardiac contusion following blunt chest trauma. Eur J Emerge Med 2006;13:373–6. 10.1097/MEJ.0b013e32801112f6 17091065

[15] Joos E Tadloc MD Inaba K Diagnosis, work-up and management of blunt cardiac injuries. Trauma 2014;16:93–8.

[16] Orliaguet G Ferjani M Riou B The heart in blunt trauma. Anesthesiology 2001;95:544–8. 11506131 10.1097/00000542-200108000-00041

[17] Inoue H Ikeda N Tsuji A An autopsy case of fatal arrhythmia induced by injuries of the atrioventricular conduction system: a case report. Med Sci Law 2004;44:353–8. 15573975 10.1258/rsmmsl.44.4.353

[18] Darok M Beham-Schmid C Gatternig R Sudden death from myocardial contusion following an isolated blunt force trauma to the chest. Int J Leg Med 2001;115:85–9. 10.1007/s004140100230 11724437

[19] El-Chami MF Nicholson W Helmy T Blunt cardiac trauma. J Emerge Med 2008;35:127–33. 10.1016/j.jemermed.2007.03.018 17976783

[20] Aykan AC Oguz AE Yildiz M Complete atrioventricular block associated with non-penetrating cardiac trauma in a 40-year-old man. J Emerge Med 2013;44:e41–3. 10.1016/j.jemermed.2011.06.067 22056546

[21] Sardu C Santamaria M Paolisso G microRNA expression changes after atrial fibrillation catheter ablation. Pharmacogenomics 2015;16:1863–77. 26554530 10.2217/pgs.15.117

[22] Hasdemir H Arslan Y Alper A Severe tricuspid regurgitation and atrioventicular block caused by blunt thoracic trauma in an elderly woman. J Emerge Med 2012;43:445–7. 10.1016/j.jemermed.2010.05.069 20851553

[23] Sardu C Carreras G Katsanos S Metabolic syndrome is associated with a poor outcome in patients affected by outflow tract premature ventricular contractions treated by catheter ablation. BMC Cardiovasc Disord 2014;14:1. 25480761 10.1186/1471-2261-14-176 PMC4364311

[24] Yıldız BŞ Astarcıoğlu MA Aladağ NB The frequency of type 2 second-degree and third-degree atrioventricular block induced by blunt chest trauma in the emergency department: a multicenter study. Ulus Travma Acil Cerrahi Derg 2015;21:193–6. 26033652 10.5505/tjtes.2015.04763

[25] Sardu C Santamaria M Rizzo MR Telemonitoring in heart failure patients treated by cardiac resynchronisation therapy with defibrillator (CRT-D): the TELECART Study. Int J Clin Pract 2016;70:569–76. 27291327 10.1111/ijcp.12823 PMC5813682

[26] Schultz JM Trunkey DD Blunt cardiac injury. Crit Care Clin 2004;20:57–70. 14979329 10.1016/s0749-0704(03)00092-7

[27] Froede R Lindsey D Steinbronn K Sudden unexpected death from cardiac concussion (commotio cordis) with unusual legal complications. J Forensic Sci 1979;24:752–6. 541638

[28] Green ED Simson LR Kellerman HH Cardiac concussion following softball blow to the chest. Ann Emerge Med 1980;9:155–7. 10.1016/s0196-0644(80)80272-1 7362107

[29] Elie M-C Blunt cardiac injury. Mt Sinai J Med 2006;73:542–52. 16568196

[30] Vaseghi M Shivkumar K The role of the autonomic nervous system in sudden cardiac death. Prog Cardiovasc Dis 2008;50:404–19. 18474284 10.1016/j.pcad.2008.01.003 PMC2752648

[31] Xhemali B Ismaili Z Matua L Sudden death from vagal inhibition. Eur Sci J 2013;9:351–7.

